# Highly homologous simian T-cell leukemia virus type 1 genome in Japanese macaques: a large cohort study

**DOI:** 10.1186/s12985-024-02434-7

**Published:** 2024-07-30

**Authors:** Kou Hiraga, Tomoya Kitamura, Madoka Kuramitsu, Megumi Murata, Kenta Tezuka, Kazu Okuma, Isao Hamaguchi, Hirofumi Akari, Takuo Mizukami

**Affiliations:** 1 Research Center for Biological Products in the Next Generation, National Institute of Infectious Diseases, Tokyo, Japan; 2 Management Department of Biosafety, Laboratory Animal, and Pathogen Bank, National Institute of Infectious Diseases, Tokyo, Japan; 3 National Institute of Animal Health, National Agriculture and Food Research Organization, Tokyo, Japan; 4 Center for the Evolutionary Origins of Human Behavior, Kyoto University, Inuyama, Aichi Japan; 5 Department of Microbiology, Faculty of Medicine, Kansai Medical University, Osaka, Japan; 6 Department of Clinical Laboratory, Subaru Health Insurance Society Ota Memorial Hospital, Gunma, Japan

**Keywords:** HTLV-1, STLV-1, Japanese macaques, Genome sequencing, Phylogeny, Apobec3G

## Abstract

**Background:**

Simian T-cell leukemia virus type 1 (STLV-1) is a retrovirus closely related to human T-cell leukemia virus type 1 (HTLV-1), the causative agent of adult T-cell leukemia (ATL). It has been shown that Japanese macaques (*Macaca fuscata,* JMs) are one of the main hosts of STLV-1 and that a high percentage of JMs (up to 60%) are infected with STLV-1; however, the molecular epidemiology of STLV-1 in JMs has not been examined.

**Methods:**

In this study, we analyzed full-length STLV-1 genome sequences obtained from 5 independent troops including a total of 68 JMs.

**Results:**

The overall nucleotide heterogeneity was 4.7%, and the heterogeneity among the troops was 2.1%, irrespective of the formation of distinct subclusters in each troop. Moreover, the heterogeneity within each troop was extremely low (>99% genome homology) compared with cases of STLV-1 in African non-human primates as well as humans. It was previously reported that frequent G-to-A single-nucleotide variants (SNVs) occur in HTLV-1 proviral genomes in both ATL patients and HTLV-1 carriers, and that a G-to-A hypermutation is associated with the cellular antiviral restriction factor, Apobec3G. Surprisingly, these SNVs were scarcely observed in the STLV-1 genomes in JMs.

**Conclusions:**

Taken together, these results indicate that STLV-1 genomes in JMs are highly homologous, at least in part due to the lack of Apobec3G-dependent G-to-A hypermutation.

**Supplementary Information:**

The online version contains supplementary material available at 10.1186/s12985-024-02434-7.

## Repositories

The STLV-1 nucleotide sequences have been submitted to the DNA Data Bank of Japan (DDBJ) with NCBI accession numbers LC490306–LC490350.

## Background

Primate T-cell leukemia virus type 1 (PTLV-1) (family *Retroviridae*; genus *Deltaretrovirus*) is an enveloped, positive-sense, single-stranded RNA virus. The deltaretrovirus contains two viral lineages: PTLV and bovine leukemia virus [1]. The PTLV lineage includes simian and human T-cell leukemia viruses (STLV and HTLV, respectively) [2, 3]. Human T-cell leukemia virus type 1 (HTLV-1) is a pathogenic virus that has spread globally to many regions, including Japan, the Caribbean, South America, and sub-Saharan Africa [4–7]. HTLV-1 causes adult T-cell leukemia/lymphoma (ATL), HTLV-1-associated myelopathy/tropical spastic paraparesis, and other inflammatory diseases in less than 5% of infected individuals [4, 7]. Evolutionary phylogenetics has shown that HTLV-1 emerged from the spread of simian T-cell leukemia virus type 1 (STLV-1) from non-human primates (NHP) to humans [8, 9]. Zoonotic transmission to humans has been confirmed in some African countries, with cases emerging after receiving severe bites from monkeys infected with STLV-1 [10].

Old World monkeys and apes are the natural hosts of STLV-1. HTLV-1 and STLV-1 share approximately 90%–95% amino acid homology, and both are approximately 60% homologous to human T-cell leukemia virus type 2 (HTLV-2) [11, 12]. Furthermore, STLV-1 infection can lead to the development of lymphoproliferative diseases [13, 14]. On the basis of these findings, it has been proposed that STLV-1-infected NHP may be suitable animal models for HTLV-1 research [15].

Japanese macaques (*Macaca fuscata,* JMs) inhabit a wide area of Japan and are known to have a high prevalence of STLV-1 infection [15–17]. It has been reported that the high prevalence may be related to frequent horizontal and mother-to-child transmission in JM [18]. Full-length genomic sequences of STLV-1 have been reported for Old World monkeys and baboons, but very limited information on sequences from JMs is available [19–22]. HTLV-1 genotypes, which are relatively well understood primate T-cell leukemia viruses, are composed of four major subtypes: Cosmopolitan subtype A, Central African subtype B, Central African/Pygmies subtype D, and Australo-Melanesian subtype C [4]. Japan is known to have a high prevalence of HTLV-1 cosmopolitan subtype A [4]. However, the genomic sequences of STLV-1 in JMs are quite different from those of HTLV-1 and STLV-1 in other NHPs [23], indicating that STLV-1 in JMs originated as a branch from a cluster that was phylogenetically distant from the Japanese subtype of HTLV-1 [23, 24]. Regional phylogenetic analyses of complete HTLV-1 subtype A genomes showed the relatively high diversity of their single-nucleotide variants (SNVs) [25, 26]. Epidemiological studies of a population within a limited area in Japan have been reported, but there have been no published phylogenetic analyses of complete genomes of STLV-1 from JMs in Japan [16]. Interestingly, STLV-1, which naturally infects African NHP, has pathogenic properties, such as inducing ATL, similar to HTLV-1 [14, 27]. There have been no reports of ATL-like leukemia in JMs infected with STLV-1. However, it is unclear what accounts for the differences in STLV-1 pathogenicity among JMs and other NHPs.

In this study, we performed a comprehensive analysis of the full-length genomic sequences of STLV-1 obtained from 5 independent troops, including a total of 68 JMs naturally infected with STLV-1. The STLV-1 genomes were analyzed for heterogeneity among individual JMs, as well as among independent troops, to compare the genomes of HTLV-1 and other STLV-1 variants.

## Methods

### Animals

Animal experiments in this study were approved by the Animal Welfare and Animal Care Committee of the Center for the Evolutionary Origins of Human Behavior (EHUB), Kyoto University (approval number: 2014–092, 2015-040, and 2016-135) and were performed in line with the Guidelines for Care and Use of Nonhuman Primates (Version 3) of the Animal Welfare and Animal Care Committee of EHUB. We followed the guidelines provided by the Guidelines for Proper Conduct of Animal Experiment and Related Activities in Academic Research Institutions [Notice No. 71 of the Ministry of Education, Culture, Sports, Science and Technology, dated June 1, 2006], which is in accordance with the recommendations of the Weatherall report titled “The use of non-human primates in research”: https://www.acmedsci.ac.uk/more/news/the-use-of-non-human-primates-in-research/. Blood samples were collected from JMs reared and bred in our open enclosures at routine health checkups under ketamine anesthesia with medetomidine, followed by the administration of the medetomidine antagonist, atipamezole, at the end of the procedure.

### Cells and genomic DNA samples

Peripheral blood mononuclear cells (PBMCs) were obtained from the blood of STLV-1 seropositive JMs bred in EHUB. To identify STLV-1 infection, particle-agglutination tests were performed on plasma samples, as previously reported [15]. The macaques were grouped into five independent troops according to the geographic regions where they lived. Genomic DNA was extracted from the PBMCs and purified using a QIAamp Blood DNA mini kit (Qiagen, Cat. No. 51104).

### Sequencing of STLV-1 genomic sequences

The full-length genomic sequences of STLV-1 were amplified as four regions by nested long PCR or PCR using a KOD-FX Neo polymerase kit (Toyobo, Code No. KFX-201), according to the manufacturer’s protocol. The thermal cycle for the first nested PCR was 94°C for 2 min, followed by 5 cycles at 98°C for 10 s, 68°C for 5 min, and 23 cycles at 98°C for 15 s, then 60°C for 15 s and 68°C for 5 min. The thermal cycle for the second nested PCR was 94°C for 2 min, followed by 45 cycles at 98°C for 15 s, 60°C for 15 s, and 68°C for 4 min. For fragment 4, the thermal cycle for the second nested PCR was 94°C for 2 min, followed by 50 cycles at 98°C for 15 s, 60°C for 15 s, and 68°C for 2 min. PCR products were purified using a QIAquick 96 PCR purification kit (Qiagen, Cat. No. 28181). The sequencing PCRs were performed using a BigDye v3.1 cycle sequencing kit (Applied Biosystems) with sequencing primers according to the manufacturer’s protocol. All the primer sequences used in the nested long PCRs and the sequencing PCRs and their positions on the STLV-1 genome are visualized in Figure S1 and listed in Tables S1 and S2. Genomic sequence analysis was performed on an Applied Biosystems 3730 DNA analyzer by Eurofin Co., Ltd., or Genewiz Co., Ltd. Contigs were assembled using the sequence-assembling software ATGC (Genetyx). Complete long terminal repeat (LTR) sequences were determined by combining consensus regions of the 5′-LTR and 3′-LTR reads, as described previously [28]. The STLV-1 nucleotide sequences have been submitted to the DNA Data Bank of Japan (DDBJ) with NCBI accession numbers LC490306–LC490350.

## Phylogenetic analysis

SNVs were extracted from the STLV-1 nucleotide sequences, and RAxML [29] with the maximum-likelihood method, and 1000 times bootstrap was used to infer the phylogenies. The topology of the phylogenetic tree was evaluated using GTRGAMMA [29].

## Results

### Genome sizes of STLV-1 in JMs

Genomic DNA extracted from PBMCs of 88 STLV-1-infected JMs from five different troops in Japan (A, H, M, T, and W) was used in this study (Fig. S2). The full-length genomic sequence of STLV-1 was determined by Sanger sequencing using DNA fragments amplified by PCRs or nested long PCRs. Resultantly, we obtained complete STLV-1 genome sequences from 68 JMs; 23 (34%, 23/68) of them shared 100% identity with the sequences of other strains. After removing the redundant sequences, we obtained full-length genome sequences of 45 unique strains of STLV-1 from JMs. These 45 sequences differed from the genome sequences of STLV-1 strains reported previously. Interestingly, the lengths of the STLV-1 genomic sequences varied by habitat area: 9,031 bps (A, M), 9,025 bps (H), 9,033 bps (T), and 9,028 bps (W) (Fig. S2B). The median overall STLV-1 genome length was 9,031 bps (Fig. S2A).

### Phylogenetic analyses of STLV-1 strains

**Fig. 1:**
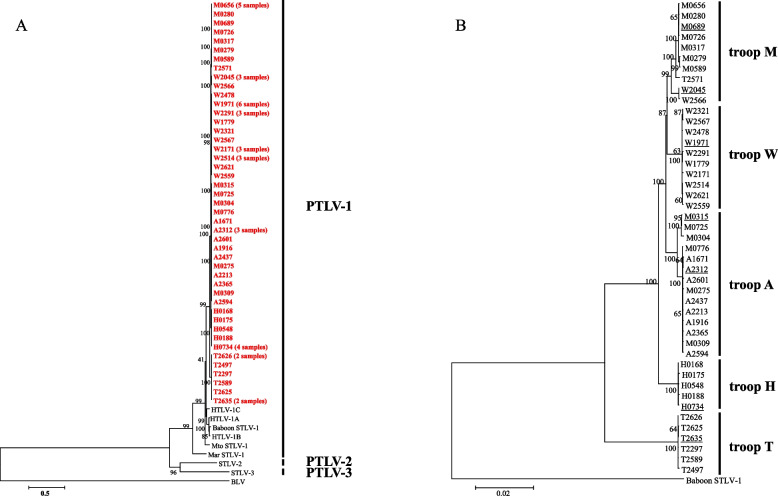
Phylogenetic trees of STLV-1 isolates from JMs. Single-nucleotide variants (SNVs) of STLV-1 were analyzed by RAxML, an algorithm that uses the maximum-likelihood method. The trees were drawn using the GTRGAMMA model. Bootstrap values are shown above and to the left of the major nodes. Scale bars indicate the number of SNVs per site. **A** Phylogenetic tree of STLV-1 and other PTLV sequences. All STLV-1 strains are shown in red. **B** Phylogenetic tree showing the relationship among STLV-1 strains from different geographical regions. Representative strains of each STLV-1 subcluster are underlined. The accession numbers of the STLV-1 sequences used to construct the trees are A1671 (LC490306), A2213 (LC490307), A2312 (LC490308), A2365 (LC490309), A2437 (LC490310), A2594 (LC490311), A2601 (LC490312), H0168 (LC490313), H0175 (LC490314), H0188 (LC490315), H0548 (LC490316), H0734 (LC490317), M0275 (LC490318), M0279 (LC490319), M0304 (LC490320), M0315 (LC490321), M0317 (LC490322), M0656 (LC490323), M0689 (LC490324), M0725 (LC490325), M0726 (LC490326), M0776 (LC490327), T2297 (LC490328), T2497 (LC490329), T2571 (LC490330), T2589 (LC490331), T2625 (LC490332), T2626 (LC490333), T2635 (LC490334), W1971 (LC490335), W2045 (LC490336), W2171 (LC490337), W2291 (LC490338), W2321 (LC490339), W2478 (LC490340), W2514 (LC490341), W2559 (LC490342), W2566 (LC490343), W2567 (LC490344), W2621 (LC490345), A1916 (LC490346), M0589 (LC490347), M0280 (LC490348), M0309 (LC490349), W1779 (LC490350), HTLV-1A (AB513134), HTLV-1B (JX507077), HTLV-1C (KF242505), baboon STLV-1 (MF621979), *Macaca tonkeana* STLV-1 (Mto STLV-1: Z46900), *Macaca arctoides* STLV-1 (Mar STLV-1: AY590142), STLV-2 (NC_001815), STLV-3 (NC_003323), and bovine leukemia virus (BLV) (AF033818)

Phylogenetic analysis of the STLV-1 genomes was conducted by maximum-likelihood analysis using RAxML. In the phylogenetic tree, the STLV-1 strains clustered together and were included in the PTLV-1 clade with HTLV-1 and STLV-1 isolated from other primates and were phylogenetically distant from PTLV-2 and PTLV-3 (Fig. 1A). Like those in other Asian macaques, such as the Toukean macaque (*Macaca tonkeana*, Mto) and the stump-tailed macaque (*Macaca arctoides*, Mar), the JM-derived STLV-1 cluster was distant from HTLV-1A, suggesting that STLV-1 and HTLV-1A diverged from a common ancestor long ago. In contrast, baboon STLV-1 clustered in close proximity to HTLV-1. Baboons, like JMs, are known to be highly susceptible to STLV-1. We generated another phylogenetic tree using the same method to examine the phylogenetic relationship among the STLV-1 strains (Fig. 1B). Interestingly, subclusters were formed by STLV-1 strains from each troop, indicating distinct regional characteristics in their genomic sequences (Fig. 1B). Surprisingly, the SNVs within the subclusters were extremely limited, with a maximum of 7 bps in H0175 (LC490314) and H0188 (LC490315).

### Nucleotide and amino acid homology between STLV-1 and other STLV-1s and HTLV-1

For comparison, we selected strains W2: W1971 (LC490335), W1: W2045 (LC490336), M1: M0689 (LC490324), M2: M0315 (LC490321), A: A2312 (LC490308), H: H0734 (LC490317), and T: T2635 (LC490334) as representative strains of each STLV-1 subcluster (Fig. 1B). The nucleotide identity of the STLV-1 isolates between geographic region T and other areas was 95.3%–95.5%, and among the other habitat areas, it was 98.5%–99.1% (Table 1). The mean homology among the subclusters was 97.9% (i.e., heterogeneity of nucleotides was 2.1%). The nucleotide homology between STLV-1 and other representative STLV-1 strains and HTLV-1 (AB513134, a representative strain of HTLV-1) was 74.7%–89.5% (Table 2).

**Table 1:**
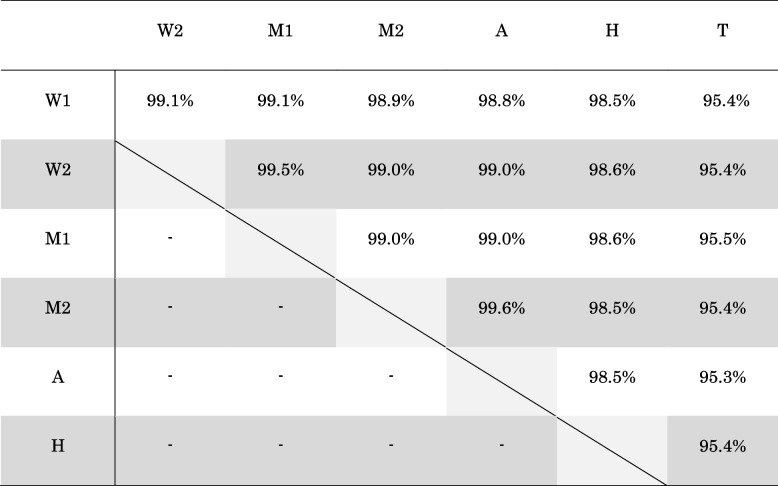
Comparison of sequence identity between geographic regions of the STLV-1 isolates

**Table 2:** Nucleotide homology of STLV-1 strains from JMs by habitat region to HTLV-1 strains

	W1	W2	M1	M2	A	H	T	Mean
HTLV-1 (AB513134)	89.5%	89.5%	89.5%	89.5%	89.4%	89.6%	89.3%	89.5%
Baboon STLV-1 (MF621979)	88.7%	88.8%	88.9%	88.8%	88.8%	88.8%	88.9%	88.8%
Mto STLV-1 (Z46900)	88.0%	88.0%	87.9%	88.0%	87.9%	88.1%	87.9%	88.0%
Mar STLV-1 (AY590142)	74.1%	75.0%	75.0%	75.0%	75.0%	73.9%	75.3%	74.7%

The amino acid homologies between the representative STLV-1 strains and previously reported PTLV strains (Table 3) were generally high (Gag 94%–95%; Pro 88%–91%; Pol 93%–95%; Env 93%–95%; Rex 83%–87%; Tax 91%–93%, SBZ 72%–79%), despite regional differences among the amino acid sequences of HTLV-1A, 1B, and 1C (Table 3). Similarly, the strains had high homology with a NHP STLV-1, namely baboon STLV-1, but relatively low homology with Mar STLV-1, which is consistent with the phylogenetic analysis of complete genomic sequences (Fig. 1A; Table 3).

**Table 3:** Amino Acid sequence comparison of STLV-1 from JMs, other PTLVs and BLV

	Protein homology between STLV-1 from JMs and other PTLVs (%)						
	Gag	Pro	Pol	Env	Rex	Tax	SBZ
HTLV-1A (AB513134)	94-95	89-91	93-94	94	83-86	92-93	72-75
HTLV-1B (JX507077)	95-96	88-89	93-94	93-94	84-87	91-93	75-78
HTLV-1C (KF242505)	95	88-90	94-95	94-95	83-87	91-92	75-79
Baboon STLV-1 (MF621979)	93	88-89	93-94	93	83-87	91-92	71-74
Mar STLV-1 (AY590142)	86-87	69-70	82	86	70-71	88	56-57
STLV-2 (NC_001815)	73-74	51	63	70	55-57	79-80	-
STLV-3 (NC_003323)	73	54-55	60	68	53-55	76-78	-
BLV (AF033818)	40	35	46	29	32	24-25	-

### Patterns of single-nucleotide variants in the STLV-1 sequences

**Fig. 2:**
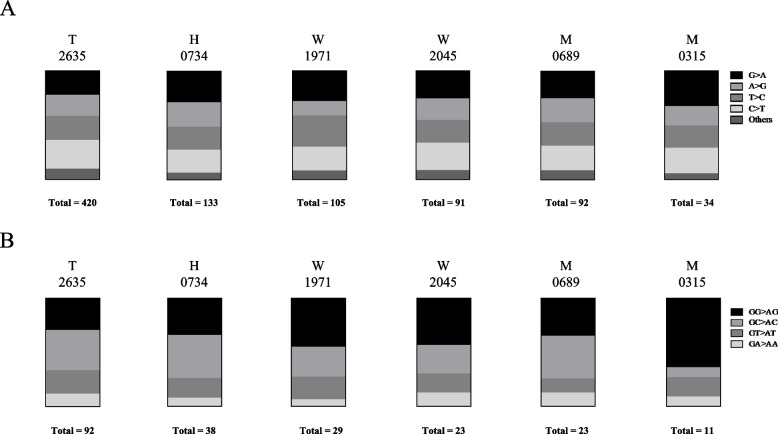
Characteristics of single-nucleotide variants in STLV-1 from JMs from five different geographical regions. **A** Types of single-nucleotide variants (SNVs) in A2312 (LC490308; a representative strain of the STLV-1) are shown as a percentage of the total number of SNVs. T2635, H0734, W1971, and M0689 were selected as representative strains from the four phylogenetic troops in Figure 2B. W2045 and M0315 were analyzed as subgroups of troops M and A, respectively. “Total” indicates the total number of SNVs in each cluster. The four major SNVs were G>A, C>T, T>C, and A>G. **B** Dinucleotide sequence context of the G>A SNVs in each troop

The types of SNVs in the STLV-1 subclusters were analyzed using the A2312 sequence (LC490308) as the standard STLV-1 sequence. The numbers of SNVs in the sequences of the six representative strains of each subcluster were 420 (T2635), 133 (H0734), 105 (W1971), 91 (W2046), 92 (M0689), and 34 (M0315). The percentages of the four most abundant SNVs were 21.9%–32.4% for G>A, 13.3%–22.6% for A>G, 20.6%–28.6% for T>C, and 21.1%–26.4% for C>T, which accounted for approximately 90% of the total SNVs in all subclusters (Fig. 2A). The average SNV rate was 26.8% for G>A, accounting for the largest proportion of SNVs in 3 of the 6 (50%) subclusters. In HTLV-1, the G-to-A hypermutation is known to be induced at the reverse transcription of the viral genome by the host enzyme Apobec3G, which contributes to host defenses against viral infection [30]. We investigated whether the host enzymes represented by the Apobec family are involved in the G>A SNV by focusing on the nucleotides downstream of the G>A site. Apobec3G shows a marked preference for editing the second C in CC sequences, producing a GG>AG substitution in the sense strand, whereas Apobec3F preferentially targets TC sequences [31, 32]. Although 63.6% of the G>A SNVs (7/11) were GG>AG in the M subcluster, a high preference for this SNV was not found in the other subclusters (Fig. 2B). This result showed no involvement of Apobec3G in STLV-1 genetic variation.

## Discussion

We obtained full-length sequences of STLV-1 from 68 JMs in five different regions of Japan. In the phylogenetic tree, the STLV-1 sequences formed a single cluster in the PTLV-1 clade and were closely related to HTLV-1 and other STLV-1 sequences. The single cluster of STLV-1 strains indicates that they evolved independently from the other PTLVs (Fig. 1A).

Among the 68 full-length STLV-1 genome sequences that we obtained, 23 (34%) were 100% identical to the sequences of other strains, which contrasts with other NHP STLV-1s. Propagation of a high frequency of completely matched STLV-1 sequences among JMs suggests that frequent horizontal transmission of a quite limited STLV-1 strain occurred within each troop. Furthermore, among the unique strains in each troop, the heterogeneity of nucleotides was extremely low (minimum 99.5% homology), indicating that the STLV-1 genomes in each troop were highly homogeneous. Conversely, the STLV-1 strains formed distinct subclusters according to their troops, and differences in genomic size were also observed among troops. These results indicate that the STLV-1 strains evolved independently within different habitat regions, maintaining highly homologous genomic sequences within an area but acquiring distinct characteristic sequences between areas (Fig. S1B). A regional phylogenetic analysis of HTLV-1 subtype A in Japan showed that the phylogenetic tree was composed of highly divergent strains, and there were no identical complete HTLV-1 sequences [26]. Previous reports of the phylogenetic characteristics of STLV-1 in NHP indicated that there are high degrees of diversity among individuals and within subgroups [33–35]. This suggests that the genomic diversity of the STLV-1 is extremely low compared with that of HTLV-1 and NHP STLV-1. In the present study, the sequence homology of STLV-1 from JM was higher than that of the previously reported STLV-1 from NHPs. STLV-1 genetic diversity may vary with primate species and their social behavior. For example, in wild chimpanzees, who have frequent contact with different primate species and often engage in aggressive group encounters, STLV-1 strains are highly diverse [33]. By contrast, in JMs, who rarely make direct contact with other primates, the distribution of STLV-1 sequences is highly conserved.

We examined the G>A ratio between the STLV-1 subclusters and, unexpectedly, did not find any distinguishing characteristics (Fig. 2). G>A SNVs have been reported to be the most common SNVs in the proviral genomes of patients with adult T-cell leukemia/lymphoma and carriers of HTLV-1, and the G>A SNV is associated with the cellular antiviral restriction factor human Apobec3G [30]. Therefore, in contrast to the correlation between HTLV-1 and Apobec3G in humans, SNVs in the STLV-1 genome may not correlate with the introduction of substitutions by simian Apobec3G in the formation of physiological subclusters. Because the proviral load in STLV-1-infected JMs is similar to that in HTLV-1-infected humans, it is possible that JMs inhibit viral replication not only through Apobec3G but also through other host factors [15]. It remains to be elucidated whether the lower diversity of the viral genome can account for the apathogenicity of STLV-1 in JMs. However, one possible explanation for the low pathogenicity of STLV-1 in JMs is that the STLV-1 genome has resistance to host Apobec3G, which helps to avoid the introduction of ATL-related nonsense mutations, such as a premature stop codon in the *Tax* gene [36].

Mutations occurred with both mother-to-child and sexual transmission, and many strains carried by females in each region can be inherited by their progeny within a certain time period [18]. However, sexual transmission of quite a limited number of strains was predominant from a long-term perspective, which contributed to the evolution of unique and extremely conserved sequences in each area. It is possible that the social structure of JMs, namely a dominant male mates with many females in the troop, provides an environment that facilitates the spread of a limited number of STLV-1 strains in an area. This hypothesis is consistent with previous results [18], which indicated that horizontal transmission was the main route of STLV-1 transmission in JMs. It was suggested that differences in the diversities of the STLV-1 and HTLV-1 genomes can be attributed to differences in the lifestyles of JMs and humans. While the observational data from our JM population may not provide sufficient information on its own to explain the mechanism of reduced STLV-1-pathogenisity in infected JMs, previous studies have also shown no evidence of pathogenicity in STLV-1-infected JMs compared with naturally infected African NHPs [14, 27]. Shichijo et al. reported that human Apobec3G stimulates activation of the transforming growth factor (TGF)-β/Smad pathway by HBZ, and this activation is associated with ATL cell proliferation, whereas the combination of SBZ and simian Apobec3g does not enhance TGF-β/Smad activity, suggesting that STLV-1 is better adapted to its simian host than is HTLV-1 [36]. It is speculated that repeated predominant horizontal transmission contributes to the acceleration of generational changes in STLV-1 strains, which might also be associated with the adaptation of STLV-1 to JMs and partially contribute to the low pathogenicity of STLV-1 in JMs.

Our results in this study showed the presence of the outlier STLV-1 in a specific troop. In fact, several JMs in troop M harbored STLV-1 with the genetic characteristics of troop A (Fig. 1B). It is well known that some JMs leave their own troops (so-called *Hanarezaru*) and occasionally immigrate to other troops [37]. It is reasonable to assume that *Hanarezaru* may introduce STLV-1 when migrating to other troops, thereby contributing to the generation of genetic outliers in a troop. Troops M and A had a relatively close geographical proximity, supporting this notion. The occurrence of these possibility will be further elucidated by an analysis of the mitochondria DNA of the troop.

STLV-1 has been used as a model virus in basic HTLV-1 research, as well as for clinical studies and the development of therapeutics and vaccines, because of their similar genetic and virological characteristics [38]. In this study, we showed that the STLV-1 provirus shares extremely high amino acid sequence homology with HTLV-1, even compared with other STLV-1s in NHP. The lowest homology (72%–79%) was in the SBZ region, but the SBZ region is known to be functionally similar to the HTLV-1 HBZ region [15].

Miura et al. [15] reported that administration of the anti-CCR4 antibody mogamulizumab to STLV-1-infected monkeys dramatically reduced proviral loads. Together, these findings indicated that STLV-1-infected JMs are a suitable model for studying asymptomatic HTLV-1 carriers and confirming the efficacy of therapeutic agents. Hasegawa et al. [39] demonstrated experimentally that the short-term cultivation of autologous PBMCs generates the Tax protein, which can serve as a therapeutic vaccine that activates cytotoxic T lymphocytes in STLV-1-infected JMs with impaired cytotoxic T lymphocytes, as seen in patients with adult T-cell leukemia/lymphoma.

## Conclusion

We have shown that STLV-1 genomes within the studied habitat areas were highly conserved and genetically highly similar to the HTLV-1 genome. Using STLV-1 as a model of HTLV-1 will strongly enhance our understanding of the basic science of STLV-1 and HTLV-1, including the pathogenetic mechanisms of HTLV-1-associated diseases, and support the development of new preventive or therapeutic strategies.

### Supplementary Information

Supplementary Material 1. 

## Abbreviations

STLV-1: Simian T-cell leukemia virus type 1
HTLV-1: Human T-cell leukemia virus type 1
ATL: Adult T-cell leukemia
JMs: Japanese macaques
SNVs: Single-nucleotide variants
DDBJ: DNA Data Bank of Japan
PTLV-1: Primate T-cell leukemia virus type 1
NHP: Non-human primates
HTLV-2: Human T-cell leukemia virus type 2
EHUB: Center for the Evolutionary Origins of Human Behavior
PBMCs: Peripheral blood mononuclear cells
LTR: Long terminal repeat
Mto: Macaca tonkeana
Mar: Macaca arctoides
BLV: Bovine leukemia virus
