# Supplementary material for: Highly homologous simian T-cell leukemia virus type 1 genome in Japanese macaques: a large cohort study
Source: Virol J. 2024 Jul 30;21:166. doi: 10.1186/s12985-024-02434-7 (PMC11290215; doi:10.1186/s12985-024-02434-7)
Supplement: Supplementary file 1 — Supplementary Material 1. [file 12985_2024_2434_MOESM1_ESM.docx]

**Supplementary information**

**Highly homologous simian T-cell leukemia virus type 1 genome in Japanese macaques: a large cohort study**

Kou Hiraga^1,2^, Tomoya Kitamura^1,3^, Madoka Kuramitsu^1*^, Megumi Murata^4^, Kenta Tezuka^1^, Kazu Okuma^5^, Isao Hamaguchi^1,6^, Hirofumi Akari^4*^, Takuo Mizukami^1^

^1^Research Center for Biological Products in the Next Generation, National Institute of Infectious Diseases, Tokyo, Japan.

^2^Management Department of Biosafety, Laboratory Animal, and Pathogen Bank, National Institute of Infectious Diseases, Tokyo, Japan.

^3^National Institute of Animal Health, National Agriculture and Food Research Organization, Tokyo, Japan

^4^Center for the Evolutionary Origins of Human Behavior, Kyoto University, Inuyama, Aichi, Japan.

^5^Department of Microbiology, Faculty of Medicine, Kansai Medical University, Osaka, Japan.

^6^Department of Clinical Laboratory, Subaru Health Insurance Society Ota Memorial Hospital, Gunma, Japan.

^*^Corresponding authors: Madoka Kuramitsu ([mkura@niid.go.jp](mailto:mkura@niid.go.jp)) and Hirofumi Akari ([akari.hirofumi.5z@kyoto-u.ac.jp](mailto:akari.hirofumi.5z@kyoto-u.ac.jp)).

**Supplementary information**


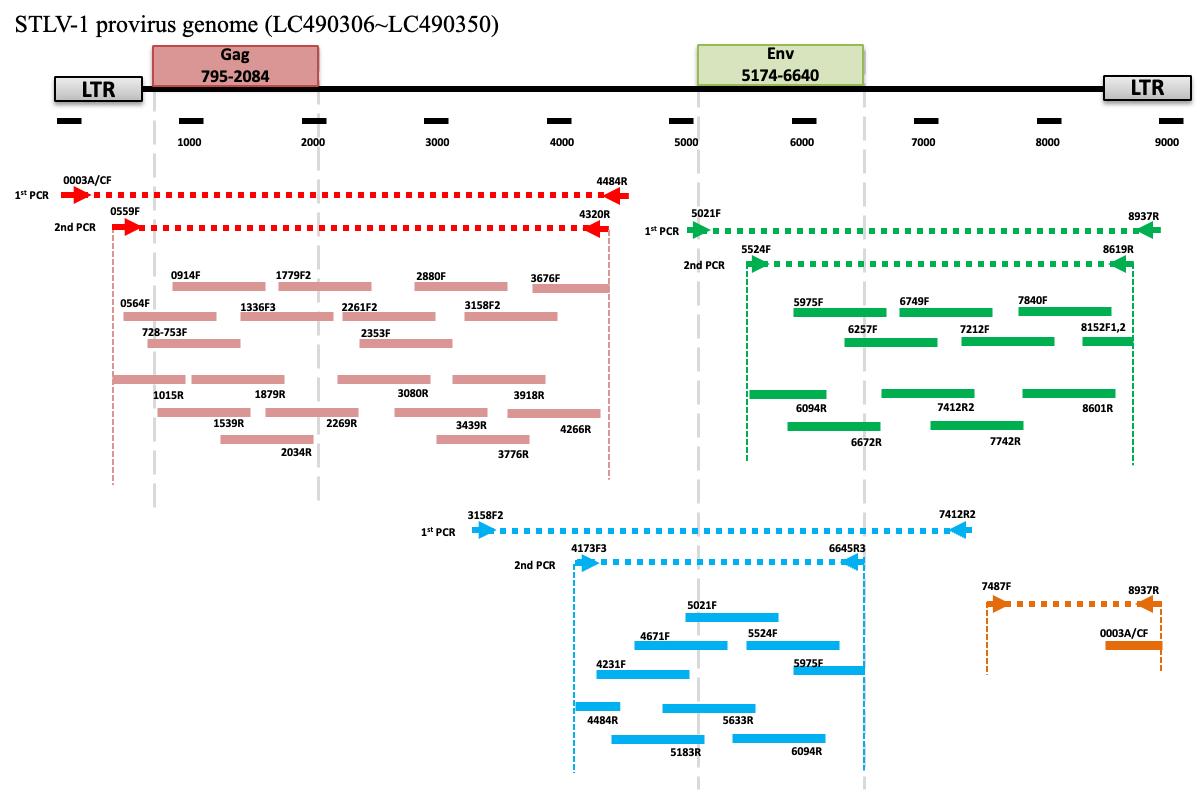


**Supplemental Figure S1. Schema of PCR and sequencing for the determination of complete STLV-1 proviral genome.**

As also described in the materials and methods and in supplemental table S1, full STLV-1 genome were amplified by 4 PCR fragments using primers indicate as arrows. Using sequencing primers indicate as bar (also listed in supplemental table S2), nucleotide sequences were read from both strands. The size of the bar is set as about 750 bases in this figure.

A

B

**Supplemental Figure S2. Differences in STLV-1 genome sizes among samples from five different geographical regions in Japan.**

Distribution of STLV-1 genome lengths in all samples from JM peripheral blood mononuclear cells (A) and samples from each geographical area in Japan (B). JM, Japanese macaques.

**Supplemental Table S1. Genomic long-PCR primers for STLV-1 full length genome sequence**

|  |  | Name | Sequence | Name | Sequence | Size | Nucleotide region equivalent to A2437 |
| --- | --- | --- | --- | --- | --- | --- | --- |
| Fragment 1 | 1st PCR | 0003AF | ATACTGACCATGAGCCCCAAA | 4484R | GCCAGATGTGGTTAGGAAGCAA | 4472 | 3-4474 |
|  |  | 0003CF | ATACTGACCATGAGCCCCACA |  |  |  |  |
|  | 2nd PCR | 0559F | TCTGACCCTGCTTGCTTAAC | 4320R | TCCGCAATGGGTGAAACTG | 3780 | 550-4329 |
| Fragment 2 | 1st PCR | 3158F2 | CCCAGGACAAAACTCARCAA | 7412R2 | CCGARCATAGTCCCCCAGAGA | 4268 | 3148-7415 |
|  | 2nd PCR | 4173F3 | CCTGCCCCGCCTACTATCRC | 6645R3 | AGGGAGGACTCGGGGTTTATAAG | 2473 | 4164-6636 |
| Fragment 3 | 1st PCR | 5021F | GGAAAGGACCACAGGARGC | 8937R | TCAGACGTGAATGAAAGGGAAAG | 3920 | 5012-8931 |
|  | 2nd PCR | 5524F | CTTAGGATGCCAATCATGGA | 8619R | TCCACGCTTTTATAGACTCCTGT | 3099 | 5515-8613 |
| Fragment 4 | - | 7487F | CCCAGAGAACCTCTAAGACCCT | 8937R | TCAGACGTGAATGAAAGGGAAAG | 1466 | 7466-8931 |

**Supplemental Table S2. Sequence primer sets corresponding to nested genomic long-PCR fragments.**

| Primers for fragment 1 | | Primers for fragment 2 | | Primers for fragment 3 | | Primers for fragment 4 | |
| --- | --- | --- | --- | --- | --- | --- | --- |
| 0564F | CCCTGCTTGCTTAACTCCACAT | 4231F | GACCCCATCTCCAGGCTCAA | 5975F | CCCCACCTGACGYTACCATT | 0003AF | ATACTGACCATGAGCCCCAAA |
| 728-753F | AGGAGAGAAGTTTAGTACACAGTTGG | 4671F | TGCCTACACTTCCCAAGACTTT | 6257F | CAAGCAATAGTCAAAAACCACAA | 0003CF | ATACTGACCATGAGCCCCACA |
| 0914F | TCCCTCCAGTTACGATTTCCA | 5021F | GGAAAGGACCACAGGARGC | 6749F | GTTCGGCCTCCAAGGAGTCT |  |  |
| 1336F3 | TTGACCCTACYGCCAAGGA | 5524F | CTTAGGATGCCAATCATGGA | 7212F | CACCAAGTCATCCACCAGTAGGT |  |  |
| 1779F2 | GGGGATATGTTGCGGGCTTG | 5975F | CCCCACCTGACGYTACCATT | 7840F | GGCCTTTCTCACCAAYGTTC |  |  |
| 2261F2 | ACAGTCCTTCCAATAGCCCTGTT | 4484R | GCCAGATGTGGTTAGGAAGCAA | 8152F1 | CAAGGCCTACCATCCCTCTT |  |  |
| 2353F | CCTTCCTGTGCTAATACGCC | 5183R | TGGTGTTGGTGGTCTTTTTCTTT | 8152F2 | CAAGGCCTATCATCCCTCCT |  |  |
| 2880F | CCCTACTTTGCCTTTACTGTC | 5633R | AGTGGAGATTAAGGCTGAGGCGTGARAC | 6094R | GGGAACAGGTGACAGRGAAAA |  |  |
| 3158F2 | CCCAGGACAAAACTCARCAA | 6094R | GGGAACAGGTGACAGRGAAAA | 6672R | AAGCAATGTGGTCGCAGTAAC |  |  |
| 3676F | TGCCAAACCATACATCATAACAT |  |  | 7412R2 | CCGARCATAGTCCCCCAGAGA |  |  |
| 1015R | AGACTAGCTAGGAGGGAATAGT |  |  | 7742R | TACATGCAGACAACGGAGTTTCC |  |  |
| 1539R | GTTGTTGGTATTCTCGCCTTAATC |  |  | 8601R | CCTGTTGTTTTATTGAGCTGTATGC |  |  |
| 1879R | CGGAAGCACGGCTGATT |  |  |  |  |  |  |
| 2034R | GGTCTAGTAGGAGGGCATCTTCCT |  |  |  |  |  |  |
| 2269R | GGCTATTGGAAGGACCGTCAT |  |  |  |  |  |  |
| 3080R | GCYAGGAGAATGTCATCCATGT |  |  |  |  |  |  |
| 3439R | GTAGCTGTACTAATGATTGA |  |  |  |  |  |  |
| 3776R | CGGTGGCTGTGGTGAAGTAGAAT |  |  |  |  |  |  |
| 3918R | AGATCCATCAGAGAATAGGCA |  |  |  |  |  |  |
| 4266R | GGCATCYGTGAGAGCGTTGA |  |  |  |  |  |  |
